# Advanced Glycation Endproducts and Bone Material Properties in Type 1 Diabetic Mice

**DOI:** 10.1371/journal.pone.0154700

**Published:** 2016-05-03

**Authors:** Mishaela R. Rubin, Eleftherios P. Paschalis, Atharva Poundarik, Gyna E. Sroga, Donald J. McMahon, Sonja Gamsjaeger, Klaus Klaushofer, Deepak Vashishth

**Affiliations:** 1 Department of Medicine, Metabolic Bone Diseases Unit, College of Physicians & Surgeons Columbia University, New York, NY, United States of America; 2 Ludwig Boltzmann Institute of Osteology at the Hanusch Hospital of WGKK, and AUVA Trauma Centre Meidling, 1st Medical Department, Hanusch Hospital, Vienna, Austria; 3 Center for Biotechnology & Interdisciplinary Studies, Department of Biomedical Engineering, Rensselaer Polytechnic Institute, Troy, NY, United States of America; INSERM - university Paris 7, FRANCE

## Abstract

Fractures, particularly at the lower extremities and hip, are a complication of diabetes. In both type 1 (T1D) and type 2 diabetes (T2D), fracture risk is disproportionately worse than that predicted from the measurement of bone mineral density. Although an explanation for this discrepancy is the presence of organic matrix abnormalities, it has not been fully elucidated how advanced glycation endproducts (AGEs) relate to bone deterioration at both the macroscopic and microscopic levels. We hypothesized that there would be a relationship between skeletal AGE levels (determined by Raman microspectroscopy at specific anatomical locations) and bone macroscopic and microscopic properties, as demonstrated by the biomechanical measures of crack growth and microindentation respectively. We found that in OVE26 mice, a transgenic model of severe early onset T1D, AGEs were increased by Raman (carboxymethyl-lysine [CML] wildtype (WT): 0.0143 ±0.0005 vs T1D: 0.0175 ±0.0002, p = 0.003) at the periosteal surface. These differences were associated with less tough bone in T1D by fracture mechanics (propagation toughness WT: 4.73 ± 0.32 vs T1D: 3.39 ± 0.24 NM/m^1/2^, p = 0.010) and by reference point indentation (indentation distance increase WT: 6.85 ± 0.44 vs T1D: 9.04 ± 0.77 μm; p = 0.043). Within T1D, higher AGEs by Raman correlated inversely with macroscopic bone toughness. These data add to the existing body of knowledge regarding AGEs and the relationship between skeletal AGEs with biomechanical indices.

## Introduction

Substantial evidence exists that in addition to the well-known complications of diabetes, such as neuropathy, nephropathy and retinopathy, increased fracture risk is an important morbidity [1]. Surprisingly, fracture risk is not fully accounted for in either type 1 (T1D) or type 2 (T2D) diabetes by measurement of bone mineral density (BMD) by dual energy X-ray absorptiometry [1]. An explanation for this discordance between fracture risk and BMD may lie in the diabetic organic bone matrix. In diabetes, the accumulation of advanced glycation endproducts (AGEs) in Type I collagen is elevated [2, 3]. AGEs are a diverse group of compounds that are generated through the non-enzymatic glycation or glycoxidation of proteins, lipids, and nucleic acids [4] with the best-studied being carboxymethyl-lysine (CML) and pentosidine [5–8]. AGEs are markedly increased in patients with diabetes [7], forming non-enzymatic cross-links within and across collagen fibers [9].

Although the effects of diabetes on collagen non-enzymatic cross linking have been characterized [5, 11] more information is needed about how AGE accumulation affects bone material at both the macroscopic and microsopic levels. We thus analyzed the bones of transgenic OVE26 mice, a model of severe, early onset progressive T1D which develops due to overexpression of calmodulin in β-pancreatic cells. This mouse model demonstrates systemic complications related to advanced T1D [10] and thus likely reflects diabetic bone disease as well. We previously showed worse propagation toughness and IDI in diabetic OVE26 mice, along with a trend for AGE levels by fluorometric analysis to be greater in diabetic OVE26 mice [11]. We now sought to investigate skeletal AGEs by Raman spectroscopic analysis at periosteal surfaces [12] in the same OVE26 mice, and their relationship with bone mechanical properties by fracture mechanics and reference point indentation.

## Materials and Methods

### Mice

Twelve female mice were studied; the characteristics of the mice have been previously published[13]. Six were wildtypes (FVB) and 6 were OVE26, a transgenic model of severe early onset T1D [FVB(Cg)-Tg(Ins2-CALM1)26Ove Tg(Cryaa-TAg)1Ove/PneJ, from the Jackson laboratory http://jaxmice.jax.org/strain/005564.html]. Diabetic OVE26 mice become hyperglycemic within 24 hours of birth due to decreased insulin production by the pancreatic β cells; insulin is 28% of control levels by 15 days. There was no difference in age (wildtype: 143 ± 3 vs OVE26 139 ± 4 days, p = 0.35). The femoral bones were dissected for analysis. Soft tissues were shaved off with an extensive cleaning procedure using a scalpel so that no muscle and soft tissue insertions remained. The bones were stored in 70% alcohol. The study was approved by the Columbia University IACUC. The research was in compliance with the guiding principles in the *Guide for the Care and Use of Laboratory Animals* [14].

### Raman Microspectroscopic Analysis

**Fig 1:**
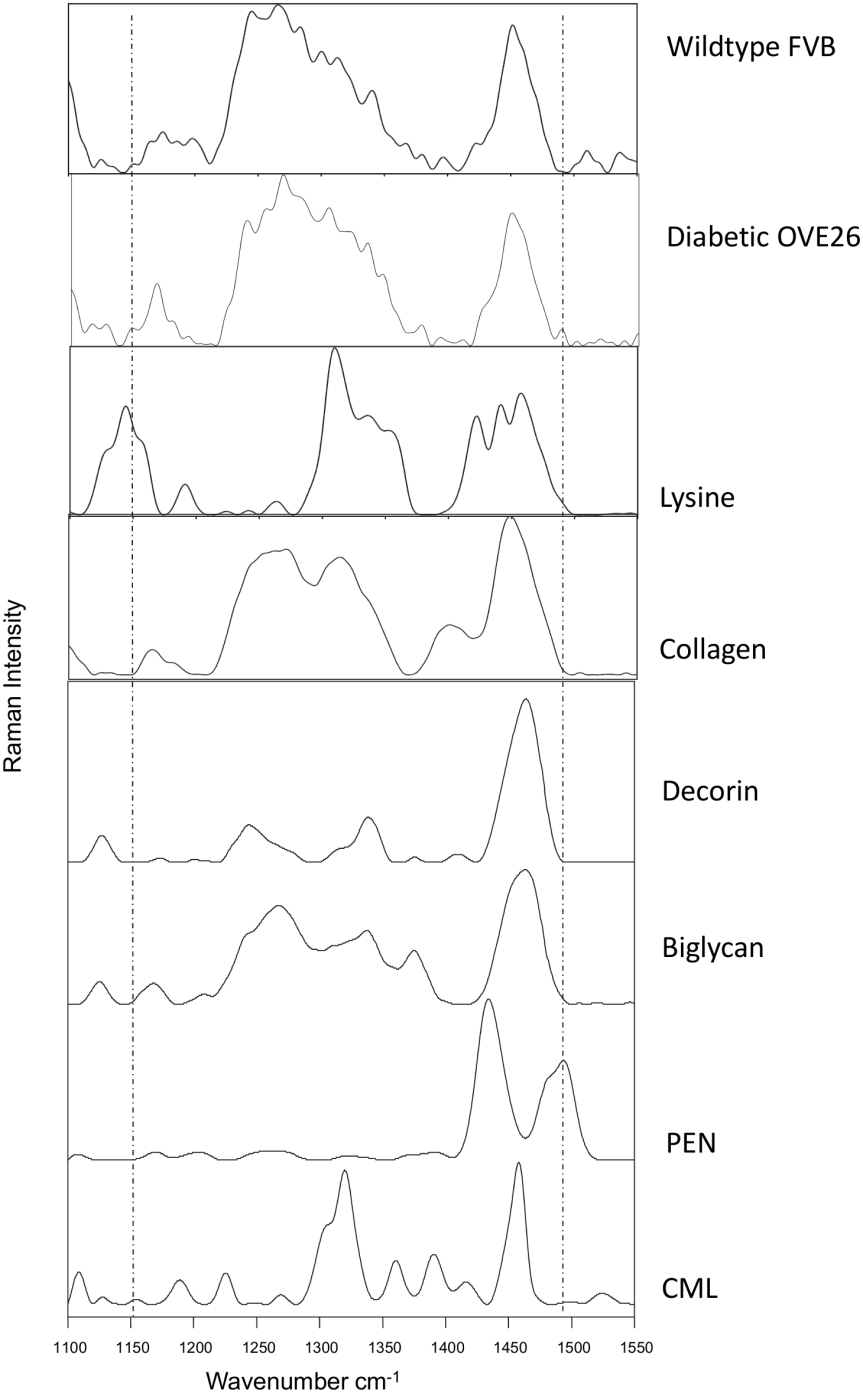
Analysis of different standard references by Raman microspectroscopy to confirm the Raman marker bands for pentosidine (PEN) and carboxymethyl-lysine (CML). The Raman measurements and spectra treatments were as described in Materials and Methods. The bone Raman spectra was obtained from the periosteal surface of a heathy and diabetic mouse. The Raman marker bands for PEN (~1495 cm^-1^) and CML (~1150 cm^-1^) are highlighted. The purified collagen Raman spectra from a human bone was kindly provided by Dr. Robins and the proteoglycan and lysine references were purchased from Sigma Aldrich. Both AGEs references (PEN and CML) were purchased from PolyPeptide Laboratories France SAS.

Mid-diaphysis periosteal surfaces of whole bones were considered. Spectra were acquired directly on the periosteal surfaces. The bones were fixed in alcohol, without any further processing before measurement. Commonly used processing methods such as dehydration with acetones and embedding in poly(methyl methacrylate) were not performed so as to minimize organic matrix constituents’ loss (such as lipids), since AGEs may anchor on oxidized proteins and lipids [8]. A Senterra (Bruker Optik GmbH) instrument was employed. The Bruker Senterra Raman spectrometer is equipped with a confocal microscope. A continuous laser beam was focused onto the sample through a Raman microscope (Olympus BX51, objective 50x) with an excitation of 785 nm (100 mW) and a lateral resolution of ~0.6 μm [15]. We focused on the surface of the sample and the collected volume was ~1x1x3 μm^3^. The Raman spectra were acquired using a thermo-electric–cooled charge-coupled device (CCD) (Bruker Optik GmbH). All data analysis was done with the Opus Ident software package (OPUS 6.5, Bruker Optik GmbH). Once acquired, the Raman spectra were baseline corrected (rubber band, 5 iterations) to account for fluorescence, and the following Raman variables were calculated as published elsewhere [15]. The mineral / matrix ratio was expressed as the ratio of the integrated areas of the *v*_2_PO_4_ (410–460 cm^−1^) to the amide III (1215–1300 cm^−1^) bands. The relative proteoglycan (PG) content was defined as the PG / organic matrix ratio, which was calculated from the ratio of the integrated areas of the proteoglycan/CH_3_ (1365–1390 cm^−1^) band representative of glycosaminoglycans [16, 17], to the amide III (1215–1300 cm^−1^) bands, respectively. The relative lipid content was expressed as the ratio of the integrated area of the lipid band ~1298 cm^-1^ / amide III [18]. The maturity / crystallinity of the bone mineral apatite crystallites was approximated from the full width at half height (FWHH) of the *v*_1_PO_4_ (930–980 cm^−1^) band, which is inversely proportional to mineral crystallinity determined by X-ray diffraction [19]. The relative content of two AGEs, namely CML (carboxymethyl-lysine) and pentosidine (PEN), was monitored as the integrated area ratio of bands at 1150 (CML) cm^-1^ or 1495 (PEN) cm^-1^ / 1450 cm^-1^ (methylene side chains (CH_2_) [20, 21], representing the AGE content normalized to the organic matrix (both collagen and non-collagenous moieties of bone [12]). The AGEs content was normalized against the CH peak ~1450 cm^-1^, as this peak reflects lipid content [18] along with collagen content to a better extent than Amide III (which is mostly representative of collagen), and both collagen and lipids can serve as substrata for AGEs formation [8]. Additional details regarding the specificity of the Raman spectral areas for CML and pentosidine are shown in Fig 1 which includes Raman spectra from a wildtype and a diabetic mouse as well as purified standards including lysine, collagen, decorin, biglycan, CML and PEN showing the specificity of the bands ~ 1150 and 1495 cm^-1^. Finally, the relative Pyd content (a major trivalent collagen cross-link) was calculated as the Raman peak height at 1660 cm^-1^ / area of the amide I (1620–1700 cm^-1^)[19, 22–26]. For each animal, 15 measurements in the area of mid-diaphysis periosteal bone were acquired; the measurements were obtained in 3 different areas with a quadrant of 5 measurements in each area. The values were averaged and the resulting value treated as a single statistical unit for the specific outcome for the specific animal.

### Fracture Mechanics

Following Raman measurements, the femurs were wrapped in saline soaked gauges and stored at -80C until testing. Fracture toughness testing done on the same femurs that were analyzed by Raman. For testing, femora from each animal were notched in the anterior mid-diaphyseal region using a slow speed diamond blade (Buehler). The anterior side was chosen to mimic natural loading conditions during three-point bending tests. Notched bones were soaked in saline for an hour prior to testing, and then tested in three-point bending, until fracture, on a custom made fixture in the displacement feedback mode (Elf 3200, EnduraTEC). A cross-head rate of 0.001mm/s was used for the tests. The resulting load-displacement curves were used to compute initiation and propagation toughness [27]. Toughness was calculated as detailed elsewhere [28], using load parameters (from load-deformation curve), structural parameters (mid-diaphyseal cortical thickness, inner and outer radii) and notch dimensions (in radians). Notch angles were determined from microCT transverse sections, using ImageJ software [29]. Initiation toughness was computed using load value at the 5% secant line (95% slope of elastic region) and propagation toughness was computed using maximum load value. Only the prefabricated notch angles were used for all calculations. While initiation toughness quantifies the resistance a material offers to the initiation of a crack, propagation toughness quantifies the resistance a material offers to crack growth and propagation.

### Reference Point Indentation

Reference point indentation was performed by the technique of Diez-Perez [30]. The technique induces microscopic fractures via cyclic indentation into a bone sample, thus providing a measure of the mechanical resistance of bone tissue to microindentation. One half of the mechanically tested femora were hydrated and used for indentation testing. Tests were performed at three points on each specimen on the anterior surface of the distal end. The total number of indentation sites was limited to 3 because of the small size of the test specimen, so as to minimize the influence of damage of previous indentations on subsequent ones (each a distance of one and half times the external probe radius away from the next). The indent closest to fracture surface was at least ~2mm away from it, to avoid any effect of the fracture toughness tests on the indentation results.

Bones were indented repeatedly for 10 indentation cycles at a frequency of 2 Hz, with a maximum force of 3 N, to induce a microfracture in the sample. A load of 3N was selected to ensure that the cortex of the fragile mice bone (~200μm thick) would not break during the indentation process. The distance by which the probe penetrated into the bone for a given load, an indicator of the resistance against the propagation of fracture, provided the indentation distance increase (IDI) which was the increase in the indentation distance in the last cycle relative to the indentation distance in the first cycle. Total indentation distance (TID) provided the total distance the test probe is inserted into the bone from touchdown to the end of the last cycle. Creep indentation distance (CID) provided the progressive indentation distance during the stable force phase of the first indentation cycle at the maximum force. For IDI, investigations have shown that a higher numerical value is consistent with worse tissue-level mechanical properties [31].

### Statistical Analysis

Wildtype FVB and diabetic OVE26 were compared with Kruskal-Wallis non-parametric tests because of the small sample size. Spearman’s correlation coefficients were used to assess the relationship between the glycation variables (CML and pentosidine), matrix variables (mineral/matrix ratio, lipids, pyridinoline, mineral maturity / crystallinity, proteoglycans) and mechanical variables (IDI, TID, CID, propagation toughness and initiation toughness). Statistical analyses were performed using PASW Statistics 18. Results are expressed as mean ± SEM; a value of p < 0.05 was considered significant.

## Results

Glucose and HbA1c levels were obtained from measurements of mice from the same colony in a previous study[13]. Blood glucose levels in that report measured at 4-week intervals beginning at 8 weeks of age remained consistently elevated until the time of sacrifice[13].

### AGE Measurement

**Fig 2:**
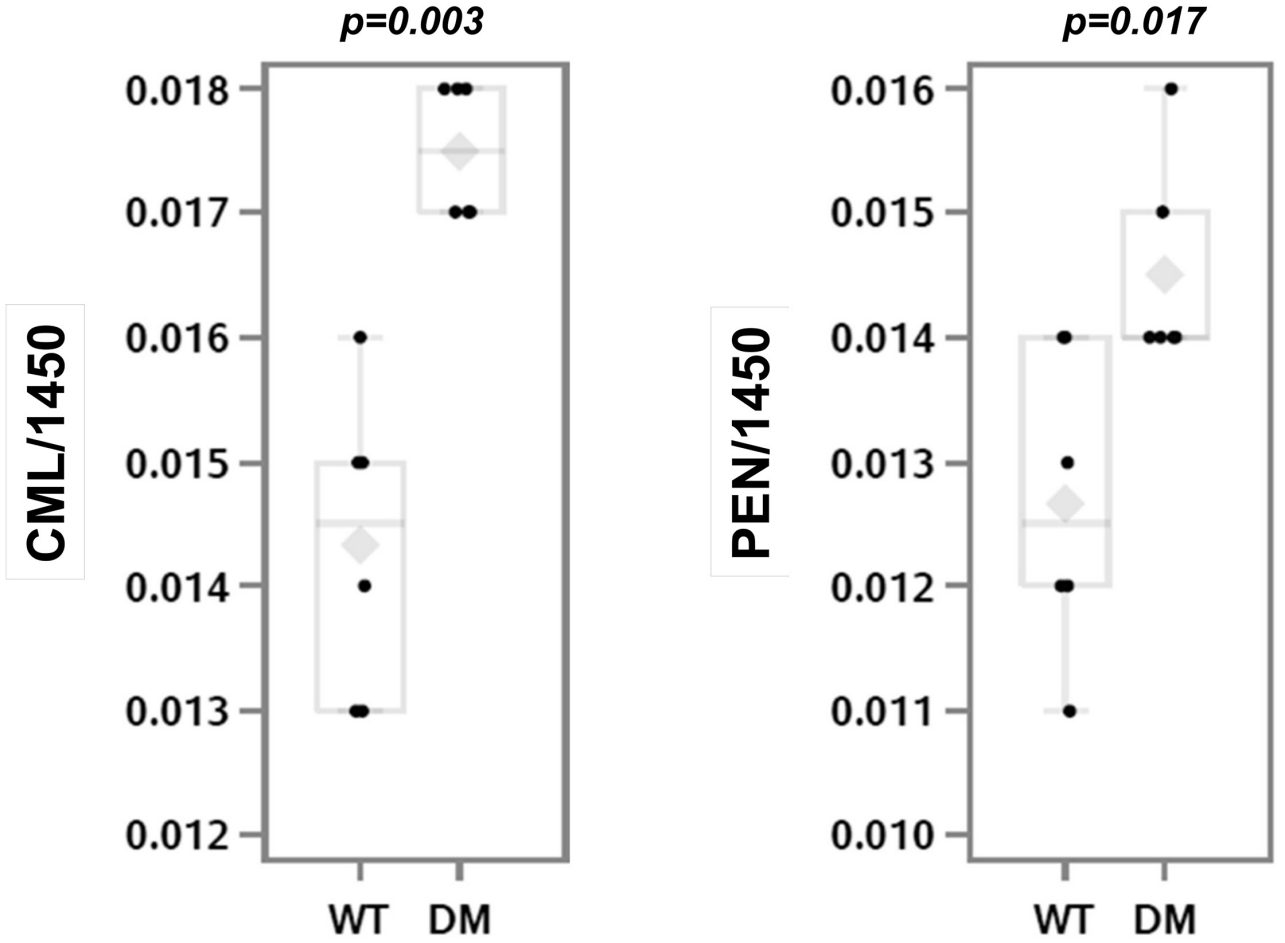
AGE measurement in the femoral bones of wildtype and diabetic mice. Raman microspectroscopic measurement of CML (panel A) and pentosidine (panel B) showed an increase in diabetic OVE26 mice (n = 6) as compared to wildtype FVB mice (n = 6); *p<0.05; **p<0.001. The mean value is indicated by the grey diamond; the median by the horizontal line; the 25% and 75% by the boxes and the 95% confidence interval by the bars.

Diabetic OVE26 mice as compared to wildtype FVB mice had greater CML (p = 0.003) and pentosidine (p = 0.017) content (Fig 2A and 2B) by Raman microspectroscopy on periosteal surfaces.

### Mineral and Organic Matrix Properties

**Fig 3:**
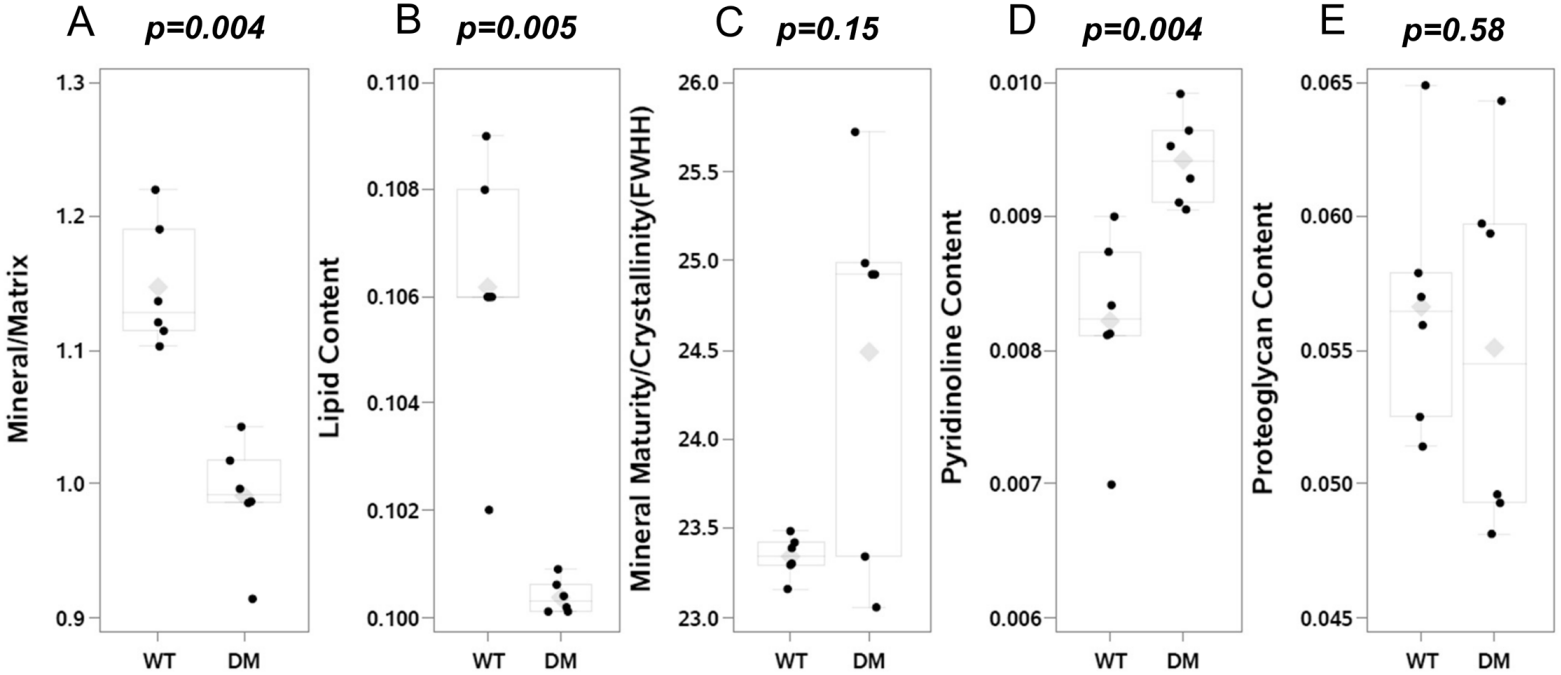
Matrix Properties by Raman Spectroscopy in femoral bones. Raman microspectroscopic measurement showed a decrease in mineral/matrix ratio [v_2_PO_4_/Amide III] (panel A) and in relative lipids content [lipids/Amide III] (panel B) in diabetic OVE26 mice (n = 6) as compared to wildtype FVB mice (n = 6). Mineral maturity/crystallinity [FWHH V_1_PO_4_] did not differ (panel C) in diabetic OVE26 mice. In contrast, relative pyridinoline content was increased in diabetic OVE26 mice (panel D), while there was no difference in relative proteoglycan content [PG/ Amide III] (panel E). The mean value is indicated by the grey diamond; the median by the horizontal line; the 25% and 75% by the boxes and the 95% confidence interval by the bars.

Diabetic OVE26 mice had decreased mineral/matrix ratio (p = 0.004; Fig 3A) and lipid content (p = 0.005; Fig 3B) as compared to wildtype FVB mice, but increased relative pyridinoline content (p = 0.004; Fig 3C). Mineral maturity/ crystallinity content did not differ between diabetic OVE26 and wildtype FVB mice (p = 0.15; Fig 3D), nor did proteoglycan content (p = 0.58; Fig 3E).

### Fracture Mechanics

**Fig 4:**
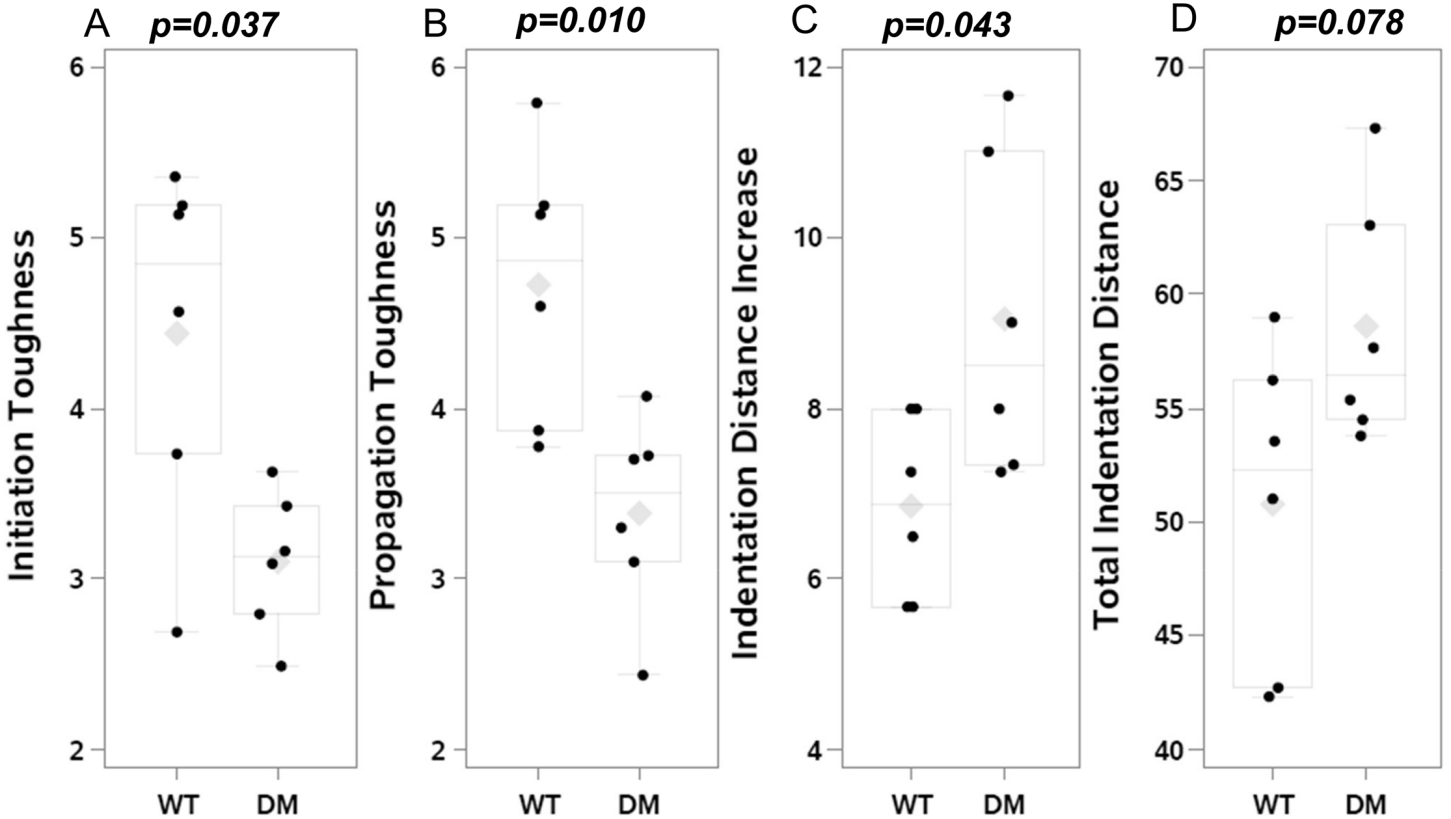
Mechanical indices in the femoral bones of wildtype and diabetic mice. Fracture toughness testing demonstrated decreased initiation (panel A) and propagation (panel B) toughness in diabetic OVE26 (n = 6) vs wildtype FVB (n = 6) mice. Reference point indentation showed increased IDI (indentation distance increase; panel C) and a tendency toward increased TID (total indentation distance; panel D). The mean value is indicated by the grey diamond; the median by the horizontal line; the 25% and 75% by the boxes and the 95% confidence interval by the bars.

In comparison to wildtype FVB mice, diabetic OVE26 mice had decreased initiation toughness (p = 0.037; Fig 4A) and propagation toughness (p = 0.010; Fig 4B).

### Reference Point Indentation

In comparison to wildtype FVB mice, diabetic OVE26 mice had greater IDI (p = 0.043; Fig 4C) and tended to have greater TID (p = 0.078; Fig 4D). CID did not differ.

### Relationships between AGEs and Mechanical and Matrix Indices

**Fig 5:**
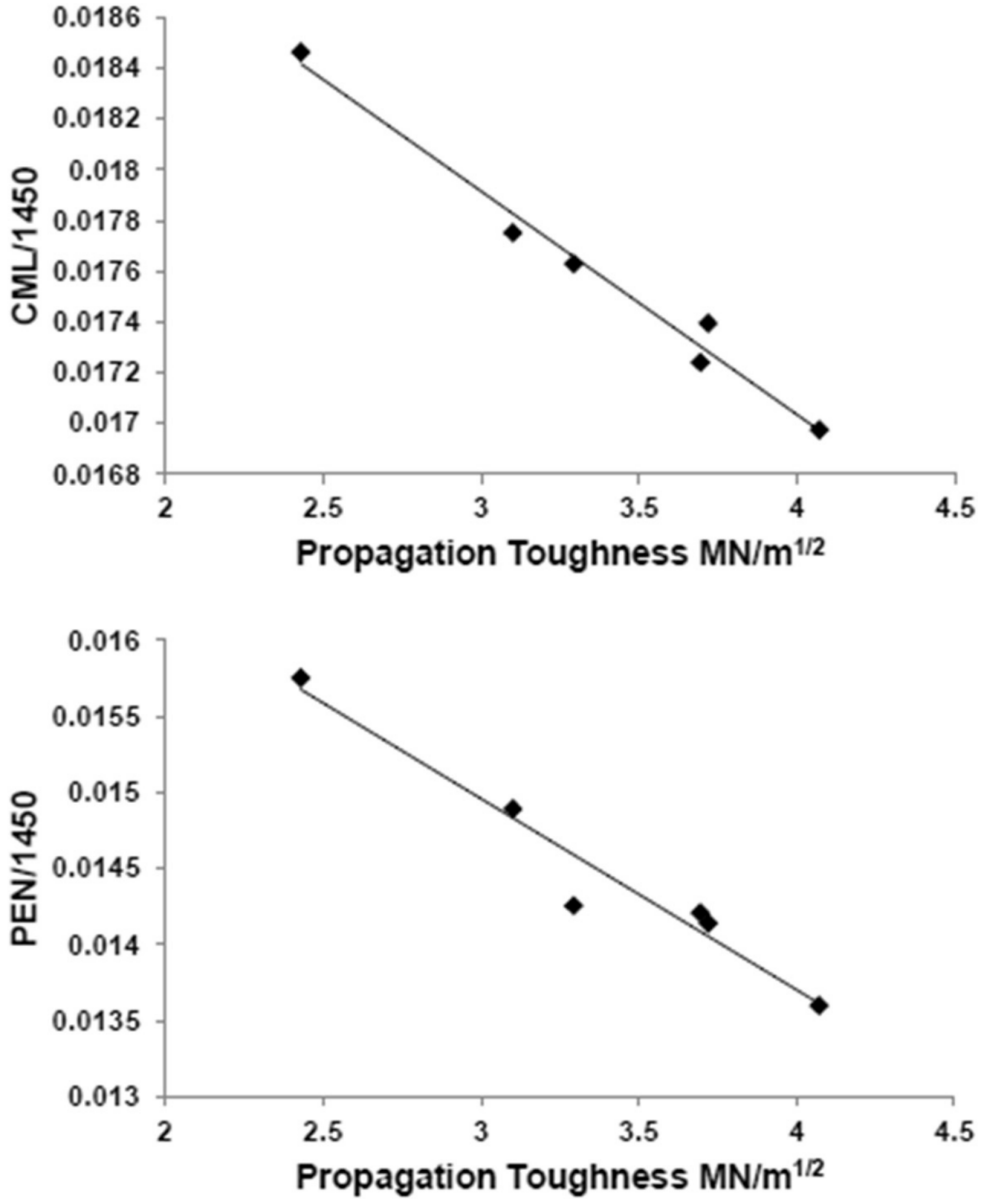
Correlation in diabetic mice between AGEs by Raman microspectroscopy and mechanical indices. Within diabetic OVE26 (n = 6) mice, CML (A) and pentosidine (B) correlated inversely with propagation toughness (r = -0.99, p<0.001 and r = -0.97, p = 0.001, respectively).

In the diabetic mice alone (n = 6), propagation toughness correlated inversely with CML (r = -0.99, p<0.001; Fig 5) and pentosidine (r = -0.97, p = 0.001). These correlations were also present in the non-diabetic mice (propagation toughness and CML: r = -0.94, p = 0.005; propagation toughness and pentosidine: r = -0.94, p = 0.005). In addition, in the diabetic mice, TID correlated inversely with mineral/matrix ratio (r = -0.86, p = 0.03); this correlation was not significant in the non-diabetic mice.

## Discussion

Bone fragility in individuals with diabetes is an issue of substantial concern [1], yet insight into the underlying causes remains limited. In contrast to postmenopausal osteoporosis, where fracture risk is linked to bone mass, bone fragility in diabetes might stem, at least in part, from deficits in organic matrix composition. Although type 1 and type 2 diabetes affect bone architecture [32, 33] and remodeling [34, 35], the accumulation of AGEs in bone organic matrix also correlates with biomechanical weakness [36]. In contrast to enzymatic cross-linking in collagen, AGE cross-links lead to more brittle bone that is less able to deform before fracturing [37]. To investigate bone AGE and mechanical indices in diabetes, we used a mouse model of severe, rapid onset T1D and performed measurements specifically designed to assess the propensity of bone matrix to fracture, at the macroscopic and microscopic levels. Our results show that diabetes- associated alterations in organic matrix composition by Raman microspectroscopy are associated with deficits in mechanical indices, as measured both by fracture mechanics and reference point indentation. These data support the concept that material abnormalities in the diabetic skeleton can be demonstrated by Raman microspectroscopy and that such abnormalities are associated with bone deterioration at multiple hierarchical levels.

Our use of Raman microspectroscopy to measure skeletal AGEs provides new information about the use of this analytical technique. Of note, the Raman measurement only reflected the AGE content in the periosteal cortical bone surface, as we focused on the surface of the sample (collected volume ~1x1x3 μm^3^). We did not perform Raman measurements further into the surface because the bone was not embedded, as this might have disturbed the lipids and thus AGEs formation [38].

Raman spectroscopy has previously been used for identification of AGEs in tissues [20, 21]. CML is a lysine derivative and in Fig 1 we have included Raman spectra from a CML reference and a lysine reference with Raman band at ~1150 cm^-1^ highlighted. This band has been described in the literature by Koenig and Sutton [39]. Collagen, a major compound in bone, has no Raman band in this area as shown in Fig 1 and in the literature [40]. Additionally in this spectral region there are no contributions from either collagen, lipids, or proteoglycans [16, 41]. Based on the purified materials used in Fig 1, a Raman band ~ 1495 cm^-1^ was used for pentosidine. Molecular structures including rings (similar but not identical to pentosidine) exhibit a Raman band ~1490 cm^-1^ [42]. This Raman band is absent in the CML reference, because the CML molecule has no ring structure.

The diabetic mice differed from the wildtype ones with regard to other Raman-measured matrix properties, including a decrease in the mineral/matrix ratio, which, unlike BMD by DXA, accounts for both the amount of mineral and organic matrix in the bone volume analyzed and is directly proportional to bending stiffness and failure moment [43]. In contrast, a report by Hammond and colleagues described an increase in mineral/matrix ratio in type 2 diabetic rats [44]. As in our study, Hammond et al. did not embed the bones, collected Raman spectra from intact diaphysis of the tibia and correlated RPI properties with Raman properties [44]. Their discrepancy in terms of mineral/matrix ratio with our data might be due to a number of reasons, including differences between skeletal effects of type 1 and type 2 diabetes [1]. We also used the v_2_PO_4_ and Amide III Raman bands, which depend solely on quantity considerations [45]. Moreover, unlike the Hammond et al study [44], we compared the periosteal bone formed at a specific anatomical location, i.e. outermost cortical, representing the newest bone mineralized matrix deposited. Accounting for bone tissue age is paramount as this ratio is very sensitive to tissue age variations[25]. In a recent report which included calcein labeling in type 1 diabetic (streptozotocin-injected *TO* Swiss) mice that underwent Fourier transform infrared microspectroscopy, Mieczkowska et al. similarly found an increase in collagen glycation and in mature collagen cross-links as compared to controls [46]. In contrast to our results, however, they did not find a decrease in the mineral/matrix ratio. This discrepancy could be explained by our lack of fluorescent labels and by the difference in mouse strains, with a potential effect of nephropathy in our model. Our findings in diabetes of increased skeletal pentosidine levels by Raman microspectroscopy are consistent with prior reports [7]. Less information is available about skeletal CML, even though this glycation product appears to be the dominant component of AGEs; on average, 30% of lysine residues present on a protein are converted to CML after glycation [47]. In a study of over 3,000 patients without diabetes the hazard ratio for hip fracture increased with each SD increase of circulating CML independent of hip BMD [48]. AGEs, as a group, decrease bone resorption by altering the structural integrity of bone matrix proteins and inhibiting the osteoclastic differentiation process [49]. This might have long-lasting effects that are similar to the “hyperglycemic metabolic memory” that has been described with AGE accumulation in other tissues [50]. In the Diabetes Control and Complications Trial (DCCT), accumulation of AGEs in skin collagen of T1D patients predicted complications decades later, regardless of subsequent improvements in glycemic control [51].

Our data suggest that in diabetes AGE accumulation co-exists with bone deterioration at both the micron level and at the fracture level. Bone resists crack growth through mechanisms that span multiple levels of hierarchy [3]. These include microcrack toughening, diffuse damage, lamellar separation, crack ligament bridges and crack deflection [3]. At the macroscopic level, we used a three point bending test to failure on notched mice bone to measure initiation and propagation toughness. Propagation toughness, which measures the ability of bone matrix to resist crack propagation and avoid catastrophic failure, was decreased in the diabetic mice bone by 29%, a level comparable to the 37.2% reduction seen *in vivo* due to three decades of aging [52]. Initiation toughness, or the resistance to the initiation of a crack, was similarly decreased. At the microscopic level, we used reference point indentation, a technique that simulates the bone fracture process at a micron level. The resultant measurement, IDI, has been established in *in vivo* studies to measure material properties of bone, with higher levels being consistent with lower fracture toughness [30, 53, 54]. We found IDI to be significantly increased in the diabetic mice, with a related increase in TID. Our results are similar to those of a clinical study in which *in vivo* reference point indentation was worse in postmenopausal women with T2D as compared to controls and correlated inversely with average HbA1c measurements over the past 10 years[55]. However, that study involved impact reference point indentation, which has a different loading mechanism than cyclic RPI and did not include skeletal AGE measurement. Importantly, there was a discrepancy in our region of interest with regard to sampling with Raman spectroscopy and with indentation, raising the possibility that the relationships we found might not have held up if tested at the same site. Nevertheless, the correlations that we found between AGEs by Raman microspectroscopy and measures of mechanical properties by fracture mechanics suggest that the extent of glycation by Raman may be a predictor of mechanical competence.

The study has a number of limitations. Our sample size was small and it is necessary to replicate these findings in a greater number of mice with concomitant glycemic measures. In addition, information about exact tissue age was lacking, since fluorescent labels were not used, which would have ensured that the age of the matrix analyzed was similar between the compared groups. It is possible that differences in periosteal formation between the groups could have influenced our results, such that diabetes was altering the chronology of matrix maturation, rather than the organic matrix composition directly. However, our measurements at the periosteal surface most likely reflected the newest bone mineralized matrix deposited in both groups. An additional limitation is that the OVE26 mice model is considered a model of diabetic nephropathy. As renal dysfunction impacts bone metabolism, it is possible that the skeletal findings were affected by diabetic nephropathy. Moreover, we did not examine whether bone material properties were homogenous throughout the entire cortical bone matrix. Our design called for preservation of the bones after Raman analysis for biomechanical testing, but further investigation of cut cross sections of bone (periosteal, center, endosteal) would address this point. Finally, our findings are specific to T1D and cannot necessarily be extrapolated to understanding the skeletal fragility associated with T2D. In T1D, insulin deficiency impairs bone formation and osteoblastogenesis [56] so that insulinopenia might have contributed to the deficits in mechanical indices that we observed. Future investigation of bone parameters in an insulin-replete high-fat diet mouse model might be more reflective of bone abnormalities in T2D.

In conclusion, our data provide new information about Raman spectroscopic measurement of skeletal AGEs and the relationship between matrix properties with macroscopic and microscopic biomechanical indices. These proof-of-concept data suggest that clinical studies of AGE measurement are important for future investigations of bone quality and fracture risk in patients with diabetes.
