# Correction: Advanced Glycation Endproducts and Bone Material Properties in Type 1 Diabetic Mice

**DOI:** 10.1371/journal.pone.0160164

**Published:** 2016-07-21

**Authors:** Mishaela R. Rubin, Eleftherios P. Paschalis, Atharva Poundarik, Grazyna E. Sroga, Donald J. McMahon, Sonja Gamsjaeger, Klaus Klaushofer, Deepak Vashishth

The fourth author’s name is spelled incorrectly. The correct name is: Grazyna E. Sroga. The correct citation is: Rubin MR, Paschalis EP, Poundarik A, Sroga GE, McMahon DJ, Gamsjaeger S, et al. (2016) Advanced Glycation Endproducts and Bone Material Properties in Type 1 Diabetic Mice. PLoS ONE 11(5): e0154700. doi:10.1371/journal.pone.0154700
